# Considerations on the Management of Zinc Phosphide Toxicity and Proposed Therapeutic Approaches

**DOI:** 10.31729/jnma.9048

**Published:** 2025-06-30

**Authors:** Foroozan Faress, Kurosh Jodaki, Leyla Abdolkarimi, Zeynab Nasri Nasrabadi, Sayed Mahdi Marashi

**Affiliations:** 1 Department of Forensic Medicine, School of Medicine, Iran University of Medical Sciences, Teharn, Iran; 2 School of Nursing and Midwifery, Shohadaye Haft e Tir Hospital, Iran University of Medical Sciences, Tehran, Iran; 3 Department of Forensic Medicine, Rajaie Cardiovascular Medical and Research Center, Iran University of Medical Sciences, Tehran, Iran; 4 Department of Pediatrics, School of Medicine, Iran University of Medical Sciences, Tehran, Iran

**Keywords:** *management*, *poisoning*, *rodenticide*, *zinc phosphide*

## Abstract

Zinc phosphide (ZnP) is a rodenticide commonly used in agriculture for pest control, but it can cause acute human poisoning, through ingestion in suicide attempts. The toxicokinetics of ZnP are not well understood, although it is believed that its primary mechanism of toxicity involves the inhibition of Complex IV of Cytochrome C Oxidase, similar to aluminum phosphide (ALP) poisoning. However, there are notable differences between the two. The rarity of acute ZnP toxicity and the misconception that it mirrors ALP poisoning have hindered the development of effective treatment strategies. This review critically examines the challenges of existing treatment protocols and proposes new approaches based on current evidence. A thorough literature review on the management of ZnP and ALP poisoning was conducted. Due to the delayed onset of symptoms in ZnP poisoning, aggressive gastrointestinal decontamination is recommended prior to toxin absorption. Furthermore, the potential for acute liver failure in early ZnP cases requires targeted treatment for hepatic injury. Since systemic absorption of phosphine can produce effects similar to ALP poisoning, existing ALP treatment protocols may also aid in managing ZnP toxicity. This study proposes a treatment protocol for ZnP poisoning, emphasizing the need for further randomized trials to validate its effectiveness.

## INTRODUCTION

Zinc phosphide (ZnP) is a rodenticide used for pest control, often ingested in suicide attempts.^1,2^

Mortality rates for ZnP poisoning are often cited as 37100%, but these figures derive from aluminum phosphide (AlP) studies.^3-5^ Limited rigorous research exists on ZnP, though Hegazy et al. reported a 1.3% mortality rate, and Trakulsrichai et al. noted 7%.^4,6^ The rarity of ZnP toxicity and unresolved questions about its toxicokinetics have slowed the development of new treatments.^7^ Most literature suggests that rapid phosphine release occurs after ZnP ingestion, leading to immediate symptoms.^4,6,8^ However, unlike AlP, ZnP poisoning may not present with acute symptoms; instead, manifestations typically appear within hours to days due to hepatic damage. This points to the unique complexities of ZnP toxicity that require further exploration.^2,7,9^

ZnP toxicity presents unique challenges, with limited understanding and few review studies compared to the more researched AlP poisoning.

## LITERATURE REVIEW

A systematic literature review was conducted to identify and synthesize existing knowledge on the management of ZnP poisoning. A comprehensive search of prominent biomedical databases, including Google Scholar, Scopus, PubMed Central, and MEDLINE, was performed using relevant keywords such as "Zinc Phosphide", "ZnP", "Aluminum Phosphide", "AlP", "Metal Phosphide", "phosphine", "toxicity", "poisoning", "management" and "treatment". Articles were meticulously reviewed to assess the scientific validity and relevance of the information presented. In cases of duplicate publications, the most recent article was prioritized to ensure the incorporation of the most up-to-date evidence. Selected articles underwent rigorous discussion among the authors, with a particular focus on elucidating the underlying pathophysiological mechanisms and identifying innovative treatment protocols. This approach enabled a thorough and comprehensive analysis of the current state of knowledge on the management of ZnP poisoning.

## MECHANISMS OF TOXICITY

It is widely accepted that the toxicity of metal phosphides is attributed to the release of phosphine upon exposure to gastrointestinal fluids following ingestion. This gas is subsequently absorbed through the intestinal mucosa and disseminated to various tissues.^1^ The mechanisms proposed for the formation of phosphine from zinc phosphide following interaction with water or acid are illustrated here:

Zn_3_P_2_ + 6H2O → 3Zn(OH)_2_ + 2PH_3_

Zn_3_P_2_ + 6[H]+ → 3Zn_2_+ + 2PH_3_

However, there are case reports of zinc phosphide poisoning in which patients remained asymptomatic despite the presence of radiopaque compounds passing through the stomach.^10^

There is limited data available on the toxicokinetics of zinc phosphide.^1^ As we know, phosphine is a highly toxic gas that is rapidly absorbed through the gastric mucosa, leading to quick onset of systemic toxicity. Consequently, it seems that the proposed formula does not adequately explain the mechanism of phosphine release from zinc phosphide. This perspective led to Marashi's hypothesis that phosphine, once released from zinc phosphide, undergoes an additional reaction to form an intermediate compound. The author cites a 1998 publication that includes an equation which may help clarify this reaction.^11,12^

PH_3_ + HCl → [Cl]^-^ + [PH_4_]^+^

The author further explains that the ionized molecule [PH4]^+^ will pass through the stomach; however, in the luminal tract, it cannot penetrate epithelial cells directly since cell membranes are permeable only to uncharged solutes. Conversely, the reductive cleavage of the [PH4]+ in basic medium of intestine yields phosphine, which can readily absorb through the intestinal mucosa.^11^ The release and absorption of phosphine gas in the intestine allows it to directly enter the portal vein and target liver cells, exerting its effects on the liver.^13^ This view is bolstered by our observations of presenting patients, although definitive proof remains absent. A hypothesis proposed that the primary cause of multiorgan dysfunction was the inhibition of Complex IV of Cytochrome C Oxidase by phosphine.^1^

The metal component of the ingested compound may also play a role in toxicity through indirect mechanisms. Measurements of metal concentrations in tissues from individuals who have died from ZnP ingestion are infrequent. For example, in one fatal case, the zinc concentration was measured at 1.16 mg/L;^14^ nevertheless, in another non-fatal case, a significantly higher zinc concentration of 14.7 mg/L was found.^15^ Thus, the relevance of these findings to toxicity has yet to be determined.

## CLINICAL MANIFESTATIONS

Approximately 30% to 60% of patients may be asymptomatic upon hospital admission. The predominant clinical features within 2 to 6 hours post-ingestion encompass burning sensations in the mouth, throat, and epigastrium, along with nausea, vomiting, and diarrhea.^2,6,16^ Furthermore, it is noteworthy that the majority of patients may exhibit normal laboratory test results upon their initial presentation.^6^

A notable concern in ZnP poisoning is the transient relief of symptoms during the second phase, which typically occurs 12 to 24 hours after ingestion. Laboratory abnormalities during this phase may present as moderate elevations in transaminases, increased levels of C-reactive protein (CRP), and metabolic acidosis.^7,13,15,17^ Due to the apparent reduction in symptoms, patients may be discharged at this stage, frequently unaware of the potentially fatal nature of the toxicity.^16^

The third phase emerges 3 to 6 days post-ingestion, marked by the onset of acute liver failure. It can progress to fulminant hepatitis, potentially leading to the patient's death.^2,16^ Additional organ dysfunction may arise, including toxic cardiomyopathy, renal failure, pulmonary edema and extensive rhabdomyolysis. The rapidity of the onset in this phase and the degree of organ involvement are likely correlated with the amount of poison consumed. Patients with the most severe cases eventually experience liver failure, leading to death.^2,16,13^

## DIAGNOSTIC TESTING

The presence of radiopaque material on an abdominal radiograph is most useful for identifying the toxin consumed. However, the lack of radiographic evidence of radiopaque material is not a reliable indicator for excluding potential toxicity.^10^ If an abdominal X-ray is negative, 90% of patients are expected to remain asymptomatic.^2^

Elevated Alanine transaminase (ALT) and Aspartate transaminase (AST) levels may result from the direct toxic effects of phosphine on liver cells, though these elevations might not be immediately apparent due to the latent absorption of phosphine. Additionally, coagulation dysfunction and an increased International normalized ratio (INR) can occur due to impaired production of coagulation factors in the liver. High anion gap metabolic acidosis, is commonly observed in severe ZnP poisoning cases. In addition, hyperglycemia may also occur and is associated with a poor prognosis.^1^

## MANAGEMENT STRATEGIES FOR ZINC PHOSPHIDE POISONING

The delayed onset of symptoms in zinc phosphide poisoning typically provides sufficient time to implement appropriate treatment measures before the clinical manifestations of poisoning appear.^1^

Gastric emptying may be beneficial if radiography indicates that a significant amount of the ingested toxin remains in the stomach.^8^ Nevertheless, in a case report by Juarez-Martinez et al., gastric lavage with coconut oil and bicarbonate was performed, but it did not prevent the onset of poisoning symptoms.^18^ While there is limited evidence from an experimental study in rats suggesting that activated charcoal may reduce the toxicity of ZnP,^19^ its chemical properties probably make it ineffective for this purpose.^20^

In general, gastric emptying is not recommended for patients with delayed presentation, as the toxin has likely passed from the stomach. However, the presence of radiopaque material in the intestinal tract may suggest that a bowel irrigation could be useful for eliminating the toxic compound.^2,10^ Whole- bowel irrigation (WBI) is accomplished by administering large volumes of an osmotically balanced polyethylene glycol electrolyte lavage solution (PEG-ELS).^21^ We cannot establish a definitive set of evidence-based protocol for the use of WBI due to the lack of clinical outcome studies on this ZnP toxicity. Parhizgar et al. documented a case of a 64-year-old man with zinc phosphide poisoning, who initially presented with normal vital signs and lab results and was treated with PEG-ELS, unexpectedly suffered sudden cardiac arrest 19 hours after admission.^22^ Despite its neutral pH, Sanaei-Zadeh suggests that using PEG-ELS for WBI may carry the risk of increased phosphine release in the intestine, given its water- soluble nature. Instead, the use of castor oil as a cathartic is suggested,^2^ although there is currently no indication for the routine use of cathartics to enhance elimination in other types of poisoning.^8^ Although Sanaei-Zadeh recommends prescribing a single dose of castor oil, since one dose is unlikely to remove all toxic compound from the intestine, it can be administered repeatedly until the radiopaque material is no longer visible on follow-up abdominal radiographs. For patients who do not show radiopaque material on the initial radiograph, it can be repeated until the odor of the poison is no longer detected in the stool. In fact, since the lack of radiographic evidence of radiopaque material is not a reliable indicator to exclude potential toxicity,^10^ and considering that only about 90% of patients with a negative abdominal X-ray remain asymptomatic,^2^ while the development of toxicity can be fatal,^4,6^ and given that repeated administration of castor oil doses is a relatively safe intervention (except for the risk of hypokalaemia due to increased gastrointestinal potassium loss), we prefer to continue gastrointestinal decontamination until the odour of the toxin in the stool resolves, even for those who doesn't have radiopaque material in their initial X-ray.

In cases of phosphine absorption resulting in systemic poisoning symptoms, no specific antidote is currently available. ZnP poisoning presents distinct challenges, such as acute liver failure and coagulopathy. Moreover, if the patient shows systemic symptoms along with metabolic acidosis, the treatment approach is similar to that for AlP poisoning.^12,16^ Several ongoing studies are exploring new treatment protocols for metal salt phosphide poisoning. One such study is examining the effects of antioxidants on the outcomes of phosphine toxicity. The results indicate that antioxidants, including N-Acetyl cysteine (NAC), L-Carnitine, Vitamin E, and Co-enzyme Q^10^, significantly reduce mortality associated with acute metal phosphide poisoning. Additionally, these antioxidants appear to decrease the necessity for intubation and mechanical ventilation.^23^

In the study by Saraf et al., which examined cases of liver failure due to ZnP poisoning, it was found that in 19 out of 41 patients who did not meet the poor prognosis according to the King's College criteria, conventional treatment along with the administration of NAC was effective in improving outcomes.^16^ A meta-analysis study investigates the effectiveness of intravenous N-acetylcysteine as an adjunctive treatment for acute aluminium phosphide poisoning. The study demonstrated a statistically significant reduction in mortality rates following the administration of intravenous NAC.^24^ Based on the positive effects observed in some case reports evaluating the use of NAC in managing ZnP poisoning,^25^ and considering the favourable cost-benefit ratio, it is recommended to prescribe NAC (300 mg/kg intravenously per day) to all symptomatic patients in this context. In their research, Bilics et al.^7^ demonstrated the effective management of ZnP poisoning accompanied by liver failure through the administration of alpha-lipoic acid (ALA), which serves both as an antioxidant and a chelating agent, alongside other supportive therapies. ALA is a naturally occurring antioxidant and coenzyme found in every cell.^26^ Historical research spanning approximately 70 years has demonstrated the therapeutic application of this treatment for conditions including alcoholic liver cirrhosis, Amanita virosa poisoning, and lead toxicity.^27-30^ The study by Bilics et al.^7^ indicated that a daily dose of 600 mg of ALA, in conjunction with NAC, can enhance liver function following ZnP poisoning. Nevertheless, the efficacy of this therapy has not been assessed in a formal case-control study.

Patients with liver dysfunction experience alterations in primary hemostasis, characterized by reduced production of all procoagulant factors except for factor VIII, decreased levels of anticoagulant proteins such as antithrombin, protein C, and protein S, and variable changes in fibrinolytic proteins.^31^ Moreover, secondary hyperfibrinolysis associated with liver dysfunction often occurs in the context of disseminated intravascular coagulation (DIC).^32^ It appears that DIC is not uncommon in cases of metal phosphide poisoning. In their study, Chugh et al., reported DIC in 6 out of 418 patients with aluminum phosphide poisoning.^33^ In their study, El Naggar and El Mahdy reported that among the 55 patients who exhibited an increase in prothrombin time after ZnP toxicity, tranexamic acid, used as an antifibrinolytic agent, and was beneficial in 16 cases.^34^ In fact, it works by competitively inhibiting the conversion of plasminogen to plasmin, thereby preventing the degradation of fibrin, which is essential for the formation of blood clots and the maintenance of hemostasis.^34^

Since acute liver failure can be a fatal complication, liver transplantation may be necessary for patients with a poor prognosis according to the King's College criteria. Saraf et al. performed liver transplants at the earliest opportunity, which appeared to be effective in saving the lives of these patients.^16^

It seems that other complications arising from the absorption of phosphine and damage to various tissues are similar to those seen in aluminum phosphide poisoning, so the treatment approach is managed in a similar manner to AlP poisoning.^2^ A significant decrease in blood pressure and the development of metabolic acidosis are two additional life- threatening complications in these patients. ^1,2,15^

Refractory hypotension often does not respond to aggressive crystalloid administration. Vasoactive agents are typically used as a secondary measure in shock management, though their effectiveness may be limited. Marashi et al. suggested that the insufficient integrity of the vascular wall, resulting from the toxic effects of phosphine and subsequent volume loss to the third space, could account for the poor response to vasoactive agents and extensive crystalloid administration.^35^ Building on this innovative concept, they proposed the use of hydroxyethyl starch for fluid resuscitation. This approach successfully led to the recovery of some patients with severe aluminium phosphide toxicity.^36,37^ However, this treatment has not been evaluated in cases of zinc phosphide poisoning. Some studies have demonstrated that the administration of angiotensin II for refractory vasodilatory shock can increase arterial pressure and has been linked to improved patient outcomes.^38,39^ Nevertheless, this treatment has not been evaluated in cases of zinc phosphide poisoning either. Further research is necessary to explore the direct effects of phosphine on vascular tissue, which could lead to the development of more effective treatments for this condition.

Another serious complication is severe metabolic acidosis. As a result, many researchers have suggested that this condition can be addressed by administering intravenous sodium bicarbonate. In this context, Jaiswal et al.^40^ conducted a detailed treatment of severe metabolic acidosis using intravenous sodium bicarbonate, monitoring base excess levels in cases of acute AlP poisoning. Although they reported a survival rate of 55%, there was no significant difference in base excess or pH levels between those who survived and those who did not. Therefore, the authors concluded that our efforts to correct acidosis aggressively do not significantly improve the prognosis. However, Sanaei-Zadeh recommends initiating treatment with sodium bicarbonate when the pH is less than 7.2 or bicarbonate levels are below 15 mEq/L, although no controlled studies have been published to support this approach.^2^ A recent study assessed the effectiveness of sodium bicarbonate in managing refractory metabolic acidosis in critically ill patients within the first 24 hours of their ICU admission. The findings revealed that sodium bicarbonate was linked to an increase in mean arterial pressure at 6 hours in patients requiring vasopressors; however, it did not show a correlation with mortality rates.^41^ Given that the administration of bicarbonate alone may not effectively improve patient prognosis, some researchers advocate focusing on fluid management and the provision of intravenous fluids in these cases.^35-37^ While these studies do not directly address ZnP toxicity, they improve our general understanding of how various treatment protocols can address acid-base disturbances. This insight could be valuable in the management of ZnP poisoning.

Although randomized clinical trials are lacking, two case reports indicate that hemodialysis and continuous renal replacement therapy may facilitate the correction of metabolic acidosis and improve the prognosis for patients who have suffered from ALP poisoning.^42,43^ Another critical aspect of managing ZnP toxicity is addressing electrolyte abnormalities, as some cardiac dysfunction may stem from these imbalances. Unlike AlP, there have been no studies assessing the importance of electrolyte abnormalities in this context. The only study addressing electrolyte disturbances in ZnP poisoning is by Trakulsrichai et al., which documented instances of hypernatremia, hyperkalemia, and hypoglycemia in affected patients.^6^ In addition to addressing metabolic acidosis, hemodialysis may also prove beneficial in rectifying electrolyte imbalances.

Oxidative stress, inhibition of cellular metabolism, and necrosis of cardiac tissue may contribute to the harmful effects of phosphine on the heart. The prevailing consensus among authors is that myocardial injury is the primary mechanism underlying cardiovascular toxicity.^44^ Therefore, ensuring proper cardiac function is crucial in patients presenting with systemic symptoms of poisoning to improve their chances of survival.

The intra-aortic balloon pump mechanically aids in establishing blood flow. Moreover, several successful case reports have proposed a new concept regarding the potential effects of digoxin and trimetazidine on improving the neurohormonal profile. Furthermore, the administration of high doses of insulin has been demonstrated to improve myocardial contractility by promoting energy production from carbohydrates and restoring calcium fluxes within cardiomyocytes.^44^ While it is crucial to maintain blood pressure in the patient, the administration of vasoactive agents may not be beneficial. Instead, it can place significant stress on a hypoperfused myocardium, often leading to terminal cardiac dysrhythmias and contributing to a serious outcome.^45^ In three distinct studies conducted from 2015 to the present, Mohan et al. documented the effective management of ALP poisoning accompanied by refractory cardiogenic shock through the use of extracorporeal membrane oxygenation (ECMO).^46-^ ^48^ ECMO is generally regarded as a treatment of last resort, employed when other therapeutic interventions have proven ineffective. As a form of extracorporeal life support, ECMO involves circulating the patient's blood outside of the body through a heart-lung machine, which removes carbon dioxide and enriches the blood with oxygen. The oxygenated blood is then returned to the patient, ensuring sufficient oxygen delivery to the tissues while allowing the heart to recover from its underlying injury.^49^ Case studies have demonstrated potential improvement in cardiac function by the fifth day following ALP toxicity,^50^ this findings offer a hopeful indication that the use of ECMO may be effective in saving patients' lives. Nevertheless, the use of ECMO carries several potential risks, including bleeding, infection, and organ damage, largely due to the significant anticoagulation required during the procedure. In the context of ZnP poisoning, the efficacy of ECMO may be limited due to the unique mechanisms of phosphine toxicity. ZnP induces cellular hypoxia through mitochondrial dysfunction, leading to multiple organ failure. As such, while ECMO can offer cardiovascular support, it may not adequately address the cellular and metabolic damage induced by ZnP. The decision to implement ECMO should be individualized, considering the patient's overall clinical status, the severity of the poisoning, and the potential risks and benefits. Moreover, The role of magnesium, hyperinsulinemic- euglycemic therapy, and the use of vasopressors such as noradrenaline and adrenaline, in addition to intravenous lipid emulsion, has been supported by studies in mitigating toxin- induced effects on myocytes.^2,23,50^

## CONCLUSION

Acute poisoning with metal phosphides is a problem predominantly observed in developing countries. In the scientific literature, zinc phosphide poisoning is not well described and is often overshadowed by references to aluminum phosphide poisoning, despite the fact that the clinical manifestations of these two poisonings are quite different. Unlike aluminum phosphide poisoning, the symptoms of zinc phosphide toxicity appear with a delay. However, if not properly treated, phosphine released from zinc phosphide can cause mitochondrial dysfunction, inhibition of cellular respiration, and a decrease in mitochondrial membrane potential, along with acute hepatic toxicity. Considering the significant time interval between poisoning and symptom onset, ZnP poisoning allows sufficient time for gastrointestinal decontamination. However, if treatment is delayed, allowing the toxic substance to accumulate, severe acute liver failure may develop. In such cases, liver transplantation can be a life-saving intervention. Additionally, clinical manifestations due to cellular respiration disturbances, such as severe metabolic acidosis and hypotension, can occur due to the effects of phosphine. These manifestations should be managed similarly to aluminum phosphide poisoning. In this study, we propose a treatment protocol for ZnP poisoning based on the current clinical evidence, primarily focusing on gastrointestinal decontamination through the administration of castor oil as a cathartic, prior to significant toxin absorption and the development of toxicity symptoms. Although this protocol is supported by existing data, further randomized trials are necessary to confirm its efficacy.
